# Tailoring the Luminescence Properties of Cs_**2**_TeCl_**6**_ Nanocrystals *via* High-Pressure and Low-Temperature Stimuli for Multiparameter Optical Manometry and Thermometry

**DOI:** 10.1021/acs.inorgchem.5c03775

**Published:** 2025-09-26

**Authors:** Zhiyu Pei, Marcin Runowski, Przemysław Woźny, Peng Du, Ning Chen

**Affiliations:** † School of Physical Science and Technology, 47862 Ningbo University, Ningbo, Zhejiang 315211, China; ‡ 467899 Adam Mickiewicz University, Faculty of Chemistry, Uniwersytetu Poznańskiego 8, Poznań 61-614, Poland

## Abstract

To search for a bifunctional luminescent platform for optical manometry and thermometry, Cs_2_TeCl_6_ nanocrystals were prepared. Excited at 456 nm, the designed nanocrystals emitted dazzling orange emission, whose fluorescence intensity, full width at half-maximum (fwhm), and decay time were sensitive to temperature. Through analyzing the temperature-dependent lifetime, the thermometric properties of the resulting nanocrystals were evaluated, resulting in a high relative sensitivity of 3.76% K^–1^. The pressure-related Raman spectra demonstrated the splendid structural stability and reversibility of the studied samples. By studying the pressure-dependent Raman modes, one knows that the maximum pressure sensitivity of the Cs_2_TeCl_6_ nanocrystals was 9.62 cm^–1^ GPa^–1^. Furthermore, as the pressure increases, a distinct spectral blue-shift was observed in the synthesized nanocrystals, contributing to the controllable luminescence at high-pressure. Through analyzing the pressure-dependent emission band centroid and fwhm, one knows that the maximum pressure sensitivities of the resultant nanocrystals were 3.54 and 4.82 nm GPa^–1^, respectively. Additionally, based on the pressure-dependent color coordinate, it was found that the maximum relative sensitivity of the designed nanocrystals was 4.62% GPa^–1^. Our findings imply that the utilization of high-pressure and low-temperature stimuli is an efficient route to regulate the luminescence properties of the Cs_2_TeCl_6_ nanocrystals.

## Graphical abstract

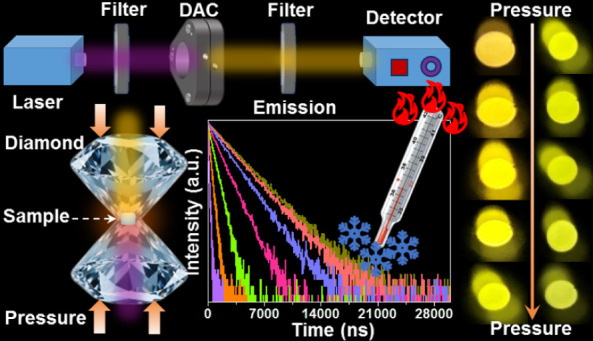

## 1 Introduction

Pressure and temperature, which act as two critical thermodynamic parameters and can impact phase structure, materials’ state, reaction kinetics, *etc*., are widely used in diverse scientific and technological fields. Thereby, the accurate detection of temperature and pressure is very important. Stimulated by these factors, considerable attention has been drawn to contactless remote measurements owing to their superiorities of rapid response, high spatial resolution, real-time feedback, *etc*.^1,2^ Up to date, for the aim of generating hydrostatic pressure to meet the requirements of industrial technology and modern scientific research, the diamond anvil cell (DAC) has been intensively employed, of which the remote optical pressure sensing is available due to the high transparency of diamonds, *i.e*., from ultraviolet to near-infrared light.^3,4^ Nowadays, through analyzing the responses of the emission band centroids of ruby (Al_2_O_3_:Cr^3+^) and SrB_4_O_7_:Sm^2+^ to pressure, the generated hydrostatic pressure in a DAC can be easily monitored. Nevertheless, these two commonly used optical manometers exhibit unsatisfactory manometric performance; *i.e*., the sensitivities of Al_2_O_3_:Cr^3+^ and SrB_4_O_7_:Sm^2+^ are 0.365 and 0.255 nm GPa^–1^, respectively.^5,6^ To solve this deficiency, researchers have developed many different kinds of luminescent materials, such as Sr_4_Al_14_O_25_:Mn^4+^ (dλ/d*p* = 1.2 nm GPa^–1^), Ca_8_Zn­(SiO_4_)_4_Cl_2_:Eu^2+^ (dλ/d*p* = 4.18 nm GPa^–1^), Li_4_SrCa­(SiO_4_)_2_:Eu^2+^ (dλ/d*p* = 5.19 nm GPa^–1^), Zn_3_Ga_2_GeO_8_:Mn^4+^ (dλ/d*p* = 0.844 nm GPa^–1^), Sr_2_[MgAl_5_N_7_]:Eu^2+^ (dλ/d*p* = 5.07 nm GPa^–1^), Ba_3_Lu­(BO_3_)_3_:Ce^3+^ (dλ/d*p* = 3.51 nm GPa^–1^), *etc*.,^7–12^ so as to build novel optical manometers, in which the pressure-related emission band centroid was adopted as the manometric parameter. Apparently, designing new luminescent materials is a facile strategy to develop highly sensitive optical manometers.

On the other hand, substantial interest has also been attracted to noncontact optical thermometry, which is based on the thermosensitive spectral characteristics, *i.e*., fluorescence intensity ratio (FIR), bandwidth, decay time, and color coordinate, of luminescent materials.^13–16^ Over the last decades, the majority of the designed optical thermometers utilized the FIR of two emission bands from rare-earth and transition metal ions, which presents a high resistance to external interferences, including doping content, excitation pump power, and other local inhomogeneities that influence sensitivity.^17,18^ Nevertheless, this method is sensitive to the light wavelength, *i.e*., light scattering and absorption effect, and thus, the temperature readout of the FIR technique is inevitably interfered with by temperature.^18,19^ Moreover, these previously developed FIR-based optical thermometers are mainly used in detecting high temperatures, and high-sensitivity low-temperature examination is still a challenge. To tackle these issues, researchers proposed lifetime-based optical thermometry, and some admirable achievements were reported. By using the decay time of Pt^4+^ as a temperature indicator, it was revealed that the maximum relative temperature sensitivity (*i.e*., *S*_r_) of the Cs_2_PtCl_6_ double perovskite reached up to 1.21% K^–1^.^20^ On the basis of the temperature-associated decay time of Sb^3+^, Zeng et al. stated that the *S*_r_ value of the Cs_2_KYbCl_6_:Sb^3+^ double perovskite was dependent on Sb^3+^ content and its maximum value was 13.6% K^–1^, allowing its application in high-temperature detection.^21^ Similar results were also found in the Er^3+^/Yb^3+^-codoped Cs_2_Ag_0.6_Na_0.4_In_0.9_Bi_0.1_Cl_6_ microcrystals, of which its *S*_r_ value was 1.40% K^–1^.^22^ Through analyzing the relation between temperature and the lifetime of Te^4+^, Wu et al. revealed that the maximum *S*_r_ value of the (TEA)_2_SnCl_6_:Te^4+^ microcrystals was 0.57% K^–1^ and it could be operated in the temperature range of 100–340 K.^23^ Furthermore, when the lifetime of the self-trapped exciton (STE) emission of the Cs_2_NaInCl_6_ microparticles was adopted as a thermometric parameter, its maximum *S*_r_ value was 3.8% K^–1^, whose operating range was 303–443 K.^24^ Evidently, the lifetime-based optical thermometers possess good thermometric properties, which can be regulated by selecting the proper host compound and doping ions. In light of these characteristics, it is appealing to develop lifetime-based optical thermometers and modify their thermometric properties.

Recently, lead-free metal halide double perovskites have been widely studied and applied in many different fields, such as optical thermometry, light-emitting diodes, optical anticounterfeiting, *etc*., regarding their good photophysical performance, structural stability, and environmental friendly features.^25–27^ It was confirmed that the luminescence properties of the Cs_2_ZrCl_6_ double perovskite can be manipulated through Pt^4+^ and Er^3+^ doping, resulting in diverse applications in anticounterfeiting, near-infrared imaging, and night vision.^28^ Shi et al., have shown that Rb_2_ZrCl_6_ nanocrystals exhibit intense broadband emission at 455 nm, enabling their feasibility for use in white light-emitting diodes.^29^ Through the introduction of Te^4+^ into the Cs_2_HfCl_6_ host lattices, Lin et al. reported that the resulting crystals not only emit intense yellow light emission with high quantum efficiency but are also suitable for solid-state lighting.^30^ A similar phenomenon was observed in other Te^4+^-doped lead-free double perovskites, such as Cs_2_ZrCl_6_ and Cs_2_SnCl_6_.^31,32^ Moreover, our recent work demonstrated a significant spectral blue shift triggered by pressure was gained in Cs_2_Ag_0.6_Na_0.4_InCl_6_:Bi^3+^ microcrystals, where the emitting color changed from yellow to blue, resulting in a high pressure sensitivity of dλ/d*p* = 112 nm GPa^–1^ with a working range up to 4 GPa.^33^ These previous research progresses suggest that lead-free metal halide double perovskites with splendid luminescence properties that can be regulated *via* doping engineering, are a promising platform for diverse applications. In spite of this, as far as we know, the utilization of high-pressure and low-temperature stimuli to adjust the luminescence characteristics of lead-free metal halide double perovskites is still limited, requiring deeper exploration of their dual-function applications in optical manometry and thermometry.

In an attempt to solve these issues, Cs_2_TeCl_6_ nanocrystals were selected as the research target and synthesized by the hydrothermal method. The crystal structure, morphological features, elemental composition, electronic structure, and luminescence properties of the designed compound were investigated in detail. By measuring the temperature-dependent emission spectra and decay curves of the resulting nanocrystals, their thermal responses were studied. Moreover, on the basis of the established relation between temperature and lifetime, the thermometric properties of the designed nanocrystals in the cryogenic temperature range of 183–303 K were explored, with a maximum *S*_r_ value of 3.76% K^–1^. Furthermore, the structural stability of the studied sample at high pressure was clarified by *in situ* high-pressure Raman spectra. Additionally, the impact of pressure on the luminescence properties of the resultant nanocrystals was characterized *via* pressure-related emission spectra in the range of 0–8.05 GPa. As pressure increased, a distinct spectral blue shift was observed in the Cs_2_TeCl_6_ nanocrystals, resulting in high-pressure sensitivities of 3.54 and 4.82 nm/GPa, respectively, when the emission band centroid and full width at half-maximum (fwhm) were employed as the manometric parameters. Notably, the prepared nanocrystals can also be used for colorific (visual) pressure sensing with a maximum relative sensitivity of 4.62% GPa^–1^. These findings imply that the utilization of high-pressure and low-temperature stimuli is an efficient route to regulate the luminescence properties of the Cs_2_TeCl_6_ nanocrystals, ensuring their promising applications in optical manometry and thermometry.

## 2 Experimental Section

### 2.1 Synthesis of Cs_2_TeCl_6_ Nanocrystals

Through using the hydrothermal method, Cs_2_TeCl_6_ nanocrystals were prepared. On the basis of the stoichiometric ratio, powders of CsCl (2 mmol) and TeCl_4_ (1 mmol), with purities of 99.5% and 99.9%, respectively, were weighed and kept in an autoclave (25 mL), to which 3 mL of HCl (37%) was added. After stirring for 10 min, the autoclave was put in an oven and heated at 200 °C for 12 h. Ultimately, the designed nanocrystals were obtained by means of the following processes, *i.e*., washing with ethanol for two times, followed by drying at 80 °C for 2 h.

### 2.2 Characterization

The crystal structure and elemental composition of the resulting nanocrystals were examined by using an X-ray diffractometer (Bruker D8 Advance, Cu Kα irradiation) and an X-ray photoelectron spectrometer (Thermo ESCALAB 250XI), respectively. For morphological characterization, a field-emission scanning electron microscope (SEM; HITACHI SU3500) equipped with an energy-dispersive X-ray spectrometer (EDS) and a transmission electron microscope (TEM; JEM-2100F, JEOL) were both utilized. *Via* utilization of a fluorescence spectrometer (Edinburgh FS5), the emission and excitation spectra of the Cs_2_TeCl_6_ nanocrystals were tested. Using a fluorescence spectrometer (Ocean Optics USB2000+), the power-dependent emission spectra of the synthesized nanocrystals were recorded. The temperature-related decay curves of the Cs_2_TeCl_6_ nanocrystals were detected with a fluorescence spectrometer (Edinburgh FSL1000). The theoretical calculation details are presented in the Supporting Information.

In order to test the *in situ* high-pressure Raman and emission spectra of the Cs_2_TeCl_6_ nanocrystal, a diamond anvil cell (DAC) apparatus was used. Here, a small amount of the Cs_2_TeCl_6_ nanocrystals was placed in a hole (≈150 μm) drilled in a stainless steel gasket with a thickness of 250 μm. In this work, the pressure transmission medium was a methanol/ethanol/water (*i.e*., volume ratio was 16:3:1) mixture, and ruby was used to monitor the generated pressure in the DAC. Excited by a 375 nm laser diode, the *in situ* high-pressure emission spectra of the Cs_2_TeCl_6_ nanocrystals were measured using a spectrometer (Andor Shamrock 500i), to which a silicon CCD camera detector was attached. Additionally, the *in situ* high-pressure Raman spectra were examined through a confocal micro-Raman system (Renishaw InVia), where a 532 nm laser diode was used as the excitation source.

## 3 Results and Discussion

It is shown in Figure 1a that the structure of the Cs_2_TeCl_6_ unit cell is composed of [CsCl_6_]^5–^ and [TeCl_6_]^2–^ octahedra, which are separated by Cs^+^. From Figure S1, one knows that the obtained diffraction peaks of the designed compounds match well with the standard cubic Cs_2_TeCl_6_ (ICSD#175772), manifesting that the final product possesses a pure phase structure, with a space group of *Fm*3̅*m* (225), which is further clarified by the Rietveld XRD refinement result, as presented in Figure 1b. Moreover, on the basis of the refinement result, one knows that the lattice parameters of the Cs_2_TeCl_6_ nanocrystals are as follows : *a* = *b* = *c* = 10.4688 Å and *V* = 1147.335 Å^3^. To explore the elemental composition of the resulting double perovskite, as well as the valence states of the elements, XPS measurement was carried out. As presented in the full XPS spectrum (see Figure 1(c)), it is clear that the elements Cs, Te, and Cl exist in the designed compounds. Particularly, the high-resolution XPS spectrum of Cs^+^ is dominated by two peaks with binding energies of 724.2 and 738.2 eV (Figure S2a), which are assigned to Cs^+^ 3d_5/2_ and Cs^+^ 3d_3/2_, respectively.^34^ Furthermore, two distinct peaks are also seen in the recorded high-resolution XPS spectrum of Te^4+^ (Figure S2b), with binding energies of 576.6 and 587.1 eV, pertaining to Te^4+^ 3d_5/2_ and Te^4+^ 3d_3/2_, respectively.^35^ In addition, for Cl^–^, its high-resolution XPS spectrum contains asymmetric peaks and it can be divided into two bands at 198.6 and 200.0 eV, corresponding to Cl^–^ 2p_3/2_ and Cl^–^ 2p_1/2_, respectively, as shown in Figure S2c.^34^ All of these results suggest that Cs_2_TeCl_6_ nanocrystals have been successfully synthesized.

**Figure 1:**
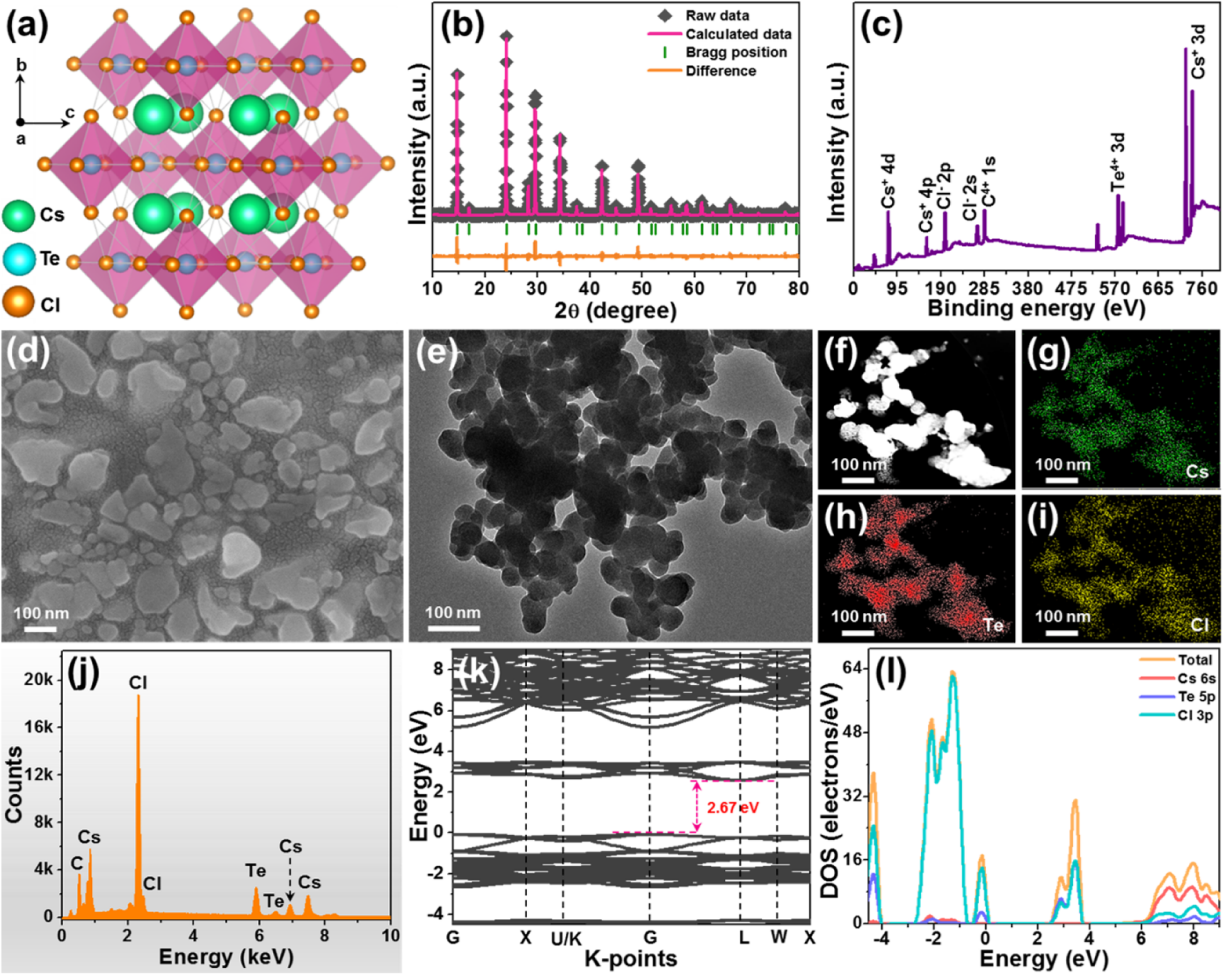
(a) 3D representation of the Cs_2_TeCl_6_ unit cell. (b) Rietveld XRD refinement pattern. (c) Full XPS survey spectrum. (d) SEM image. (e) TEM image. (f–i) EDS-based elemental mapping. (j) EDX spectrum. (k) Calculated band structure and (l) DOS of the Cs_2_TeCl_6_ material.

To analyze the morphological characteristics of the resulting double perovskite, measurements using SEM and TEM were performed. Both the SEM and TEM images indicate that the final products are composed of nanosized particles, as depicted in Figure 1d,e, respectively. Moreover, the elemental mapping results demonstrate that the elements in the studied samples are distributed equally throughout the entire nanoparticles, as illustrated in Figure 1f–i. Furthermore, the EDS spectrum shown in Figure 1j also implies that the developed compound is made up of Cs, Te, and Cl elements, which coincides well with the results obtained from the XPS spectrum. By utilization of density functional theory calculations, the electronic structure of the Cs_2_TeCl_6_ nanocrystal was analyzed. As disclosed, the Cs_2_TeCl_6_ nanocrystal exhibits an indirect band gap of 2.67 eV (see Figure 1k), in which the top of the valence band is located at the Γ point, while the bottom of the conduction band is situated at the L point. Additionally, it is demonstrated in Figure 1l that both the conduction band and valence band are contributed by Te 5p and Cl 3p orbitals. The diffuse reflectance spectrum of the Cs_2_TeCl_6_ nanocrystal was recorded and is presented in Figure S3a. It is found that the studied sample exhibits intense absorption in the range of 300–525 nm, which is assigned to the overlapped ^1^S_0_ → ^1^P_1_ and ^1^S_0_ → ^3^P_1_ transitions of Te^4+^.^34–36^ According to previous reports, one knows that the optical band gap (*i.e*., *E*_g_) of the semiconductor can be roughly estimated from the diffuse reflectance spectrum *via* the following functions:^4,37^

1 $$F\left(R\right)={\left(1-R\right)}^{2}/2R$$

2 $$hvF\left(R\right)=A{\left(hv-E_{g}\right)}^{n}$$

where the reflectance constant is labeled by *R*, *A* is a constant, *hv* stands for the photon energy, and the *n* value is determined by the category of semiconductor. Since Cs_2_TeCl_6_ pertains to the indirect transition semiconductor, its *n* value is 2. Thereby, from the plot of [*hvF*(*R*)]^1/2^ vs. *hv* (see Figure S3b), one knows that the *E*_g_ value of the Cs_2_TeCl_6_ nanocrystals is 2.49 eV, which is in good accordance with the theoretical value.

To investigate the luminescence features of the studied sample, the emission and excitation spectra of the Cs_2_TeCl_6_ nanocrystals were examined and are presented in Figure 2a. When the monitoring wavelength is 596 nm, the recorded excitation spectrum consists of a broad band, which can be divided into two parts, *i.e*., the bands in the range of 300–375 and 375–500 nm, originating from the ^1^S_0_ → ^1^P_1_ and ^1^S_0_ → ^3^P_1_ transitions of Te^4+^, respectively.^30,31^ Excited at 456 nm, the emission spectrum of the Cs_2_TeCl_6_ double perovskite consists of a strong broad band with a large Stokes shift of 119 nm (Figure 2a), arising from the recombination of the STE triggered by the strong Jahn–Teller distortion of the [TeCl_6_]^2–^ octahedron.^30,31^ For the aim of comprehending the origin of the observed emission in the final product, the power density-dependent emission spectra of the Cs_2_TeCl_6_ nanocrystals were tested and are presented in Figure 2b. As the excitation power density increases, a gradual increment is gained in the fluorescence intensity without the saturation phenomenon occurring, where the relation between the excitation power density and fluorescence intensity is linear (see Figure 2c), ruling out the emission related to the permanent defects. Moreover, when the monitoring and excitation wavelengths are 596 and 456 nm, respectively, one knows that the decay time of the Cs_2_TeCl_6_ nanocrystals is 109.5 ns, as displayed in Figure S4. It is well-known that the lifetimes of the excited states of Te^4+^ should be within the microsecond or millisecond range,^38^ which is inconsistent with that of the Cs_2_TeCl_6_ nanocrystals. Considering the unique characteristics, *i.e*., broad band emission, large Stokes shift, and short decay time of the observed emission, *etc*., it can be concluded that the generated emission arises from the recombination of STE.

**Figure 2:**
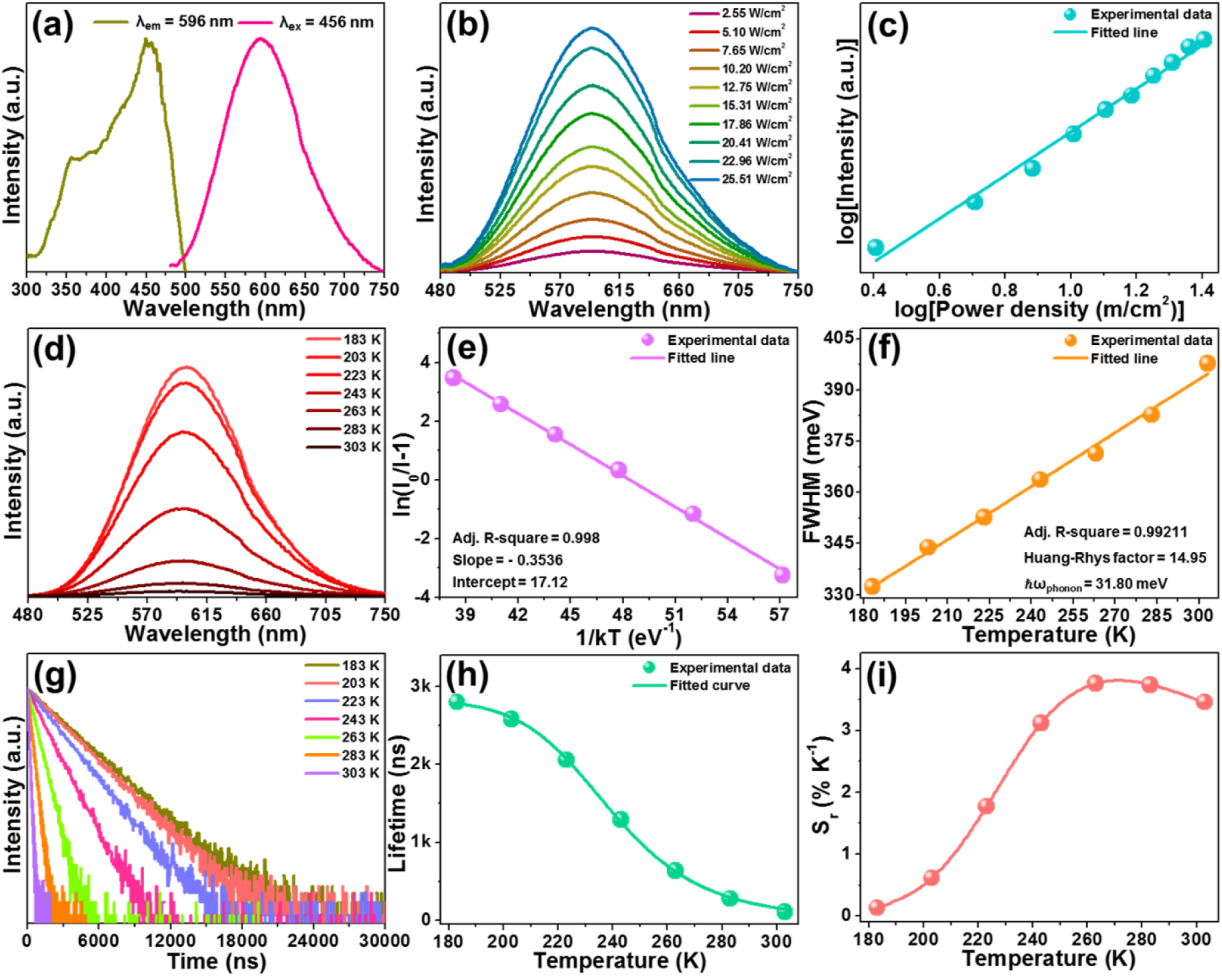
(a) Excitation and emission spectra of Cs_2_TeCl_6_ nanocrystals. Power density-dependent (b) emission spectra and (c) fluorescence intensity of the Cs_2_TeCl_6_ nanocrystals. (d) Temperature-related emission spectra of the Cs_2_TeCl_6_ nanocrystals. (e) Plot of ln­(*I*_0_/*I* – 1) vs. 1/*kT* for the Cs_2_TeCl_6_ nanocrystals. (f) Dependence of fwhm on temperature. (g) Decay curves, (h) lifetime, and (i) *S*_r_ value of the Cs_2_TeCl_6_ nanocrystals as a function of temperature.

In an attempt to study the STE dynamics and exciton–phonon interaction in the Cs_2_TeCl_6_ nanocrystals, its temperature-related emission spectra were measured using 456 nm excitation. As demonstrated (Figure 2d), the fluorescence intensity decreases rapidly as the temperature rises , owing to heat-assisted nonradiative recombination, which is beneficial for its application in optical thermometry. Moreover, the involved activation energy (*i.e*., Δ*E*) was also analyzed using the following function:^24,39^

3 $$I\left(T\right)=\frac{I_{0}}{1+A\exp\left(-\Delta E/kT\right)}$$

where the integrated emission intensities at measured temperature *T* and *T* = 0 K are denoted by *I*(*T*) and *I*_0_, respectively, *A* represents the constant, and *k* describes the Boltzmann constant. Consequently, the Δ*E* value is calculated to be 353.6 meV, as illustrated in Figure 2e, which is larger than values for previously reported luminescent materials doped with Te^4+^.^24,28^ In general, the large Δ*E* value is beneficial for promoting the photogenerated excitons to resist thermal decomposition, contributing to strong emission at room temperature.^40^ Aside from the declined luminescence, the full-width at half-maximum (fwhm) of the STE emission is found to increase monotonously with the applied temperature range (*i.e*., 183–303 K), which is assigned to enhanced electron–phonon interaction during the heating process (see Figure 2f). In light of previous reports,^24,30^ one knows that the electron–phonon coupling strength can be described *via* utilizing the Huang–Rhys factor (*S*) and phonon energy (h̅ω_phonon_), which can be evaluated by investigating the temperature-dependent fwhm, as presented below:^24,30^

4 $$\mathrm{fwhm}=2.36\sqrt{S}\hbar \omega_{\mathrm{phonon}}\sqrt{\coth\left(\frac{\hbar \omega_{\mathrm{phonon}}}{2kT}\right)}$$

Through fitting the experimental data, it is evident that the *S* value of the Cs_2_TeCl_6_ nanocrystals is 14.95, which is much larger than 5, manifesting that the electron–phonon coupling in the designed compound is strong.^41^

Considering practical applications, the resulting compounds are expected to present good structural stability under extreme conditions. From the temperature-dependent XRD patterns in the range of 100–523 K (see Figure S5), one achieves that these recorded diffraction peaks are independent of temperature and they are in accordance with the standard cubic Cs_2_TeCl_6_ (ICSD#175772), implying that the designed nanocrystals possess good structural stability at both low and high temperatures, which allows their feasibility in temperature sensing. To explore the feasibility of the resulting sample for optical thermometry, the temperature-dependent decay curves of the Cs_2_TeCl_6_ nanocrystals were recorded and are depicted in Figure 2g. Here, all of these obtained decay curves can be fitted to a double-exponential expression, as defined below:

5 $$I\left(t\right)=I_{0}+A_{1}\exp\left(-t/\tau_{1}\right)+A_{2}\exp\left(-t/\tau_{2}\right)$$

where the integrated fluorescence intensities at time *t* and *t* = 0 are described by *I*(*t*) and *I*_0_, respectively, *A*_1_ and *A*_2_ are the fitting constants, τ_1_ and τ_2_ are the lifetimes. Besides, with the aid of the following function, the average decay time (τ_avg_) can be obtained:

6 $$\tau_{\mathrm{avg}}=\frac{A_{1}\tau_{1}^{2}+A_{2}\tau_{2}^{2}}{A_{1}\tau_{1}+A_{2}\tau_{2}}$$

Evidently, with increasing temperature (183–303 K), the τ_avg_ value decreases rapidly from 2800 to 109.5 ns (see Figure 2h), allowing its application in optical temperature sensing. Note that the relation between the lifetime and temperature conforms to the following equation:^21,42^

7 $$\tau =\frac{\tau_{0}}{1+A\exp\left(-\Delta E/kT\right)}$$

where the excited state lifetimes associated with the observed emission at temperature *T* and *T* = 0 K are labeled by τ and τ_0_, respectively, *A* is a constant, and Δ*E* is attributed to the activation energy. On the basis of the experimental data, we found that the relation between lifetime and temperature can be expressed as τ = 2813.21/[1 + 1.16 × 10^6^ exp­(−3343.26/*T*)] (Figure 2h). For the sake of quantifying the thermometric properties of the developed double perovskite, the temperature-dependent *S*_r_ value was estimated through the following formula:^21,42^

8 $$S_{r}=\left|\frac{1}{\tau }\frac{d\tau }{dT}\right|\times 100\%=\left|\frac{A\exp\left(-\Delta E/kT\right)}{1+A\exp\left(-\Delta E/kT\right)}\times \frac{\Delta E}{kT^{2}}\right|\times 100\%$$

The calculated *S*_r_ values with the applied temperature range of 183–303 K are presented in Figure 2i. It is found that the *S*_r_ value increases as the temperature rises , achieving its maximum value of 3.76% K^–1^ at 263 K. Interestingly, the determined *S*_r_ value is much higher than that of the majority of previously developed optical thermometers operating in the kinetic mode (*i.e*., using the lifetime as the thermometric parameter), such as Cs_2_PtCl_6_ (*S*_r_ = 1.21% K^–1^), Cs_2_Ag_0.6_Na_0.4_In_0.9_Bi_0.1_Cl_6_:Er^3+^/Yb^3+^ (*S*_r_ = 1.40% K^–1^), (TEA)_2_SnCl_6_:Te^4+^ (*S*_r_ = 0.57% K^–1^), Cs_2_NaInCl_6_ (*S*_r_ = 3.8% K^–1^), Cs_2_NaInCl_6_:Sb^3+^/Sm^3+^ (*S*_r_ = 3.02% K^–1^), CaMgGe_2_O_6_:Cr^3+^ (*S*_r_ = 2.5% K^–1^), and so forth,^20,22–24,42,43^ implying that the Cs_2_TeCl_6_ nanocrystals can be effectively used for monitoring temperature in the mild, low-temperature, and cryogenic range by using the lifetime as the thermometric parameter.

In order to clarify the structural stability of the designed compound at high pressure, the pressure-dependent Raman spectra of the Cs_2_TeCl_6_ nanocrystals were measured and are presented in Figure 3a. At ambient conditions, the recorded Raman spectrum consists of three bands at around 138, 239.5, and 285 cm^–1^, which are assigned to the bending T_2g_ mode, asymmetric stretching E_g_ mode, and symmetric stretching A_1g_ mode, respectively.^25,36^ As pressure increases from 1 atm to 8.17 GPa, the Raman peaks do not vanish, and the extra peaks from potential secondary structure do not appear, implying that the Cs_2_TeCl_6_ nanocrystals possess good structural stability within the applied high-pressure range. Nevertheless, these Raman bands gradually shift to higher wavenumbers (*i.e*., higher energy) throughout the whole compression process, resulting from the shortened bonds at high pressure. Importantly, when the pressure decreases (*i.e*., decompression process), it can be seen that the Raman peaks shift back to lower wavenumbers (Figure 3b), suggesting that the resulting nanocrystals have good structural stability, *i.e*., full reversibility of the observed pressure-induced effects. Notably, the relations between the Raman band centroids and pressure are linear, with the shift rates of the T_2g_, E_g_, and A_1g_ Raman modes determined to be 7.35, 9.62, and 7.16 cm^–1^ GPa^–1^, respectively, as illustrated in Figure 3c–e. These results manifest that the Cs_2_TeCl_6_ nanocrystals with high structural stability and reversibility during the compression and decompression processes are suitable for high-pressure environments. Notably, compared with other luminescent materials that were developed for optical manometers, the Cs_2_TeCl_6_ nanocrystals possess the highest Raman mode shift rate, as presented in Figure 3f. Inspired by this feature, optical manometry can be realized in the designed nanocrystals by using the Raman mode as the manometric parameter, with its maximum pressure sensitivity reaching up to 9.62 cm^–1^ GPa^–1^.

**Figure 3:**
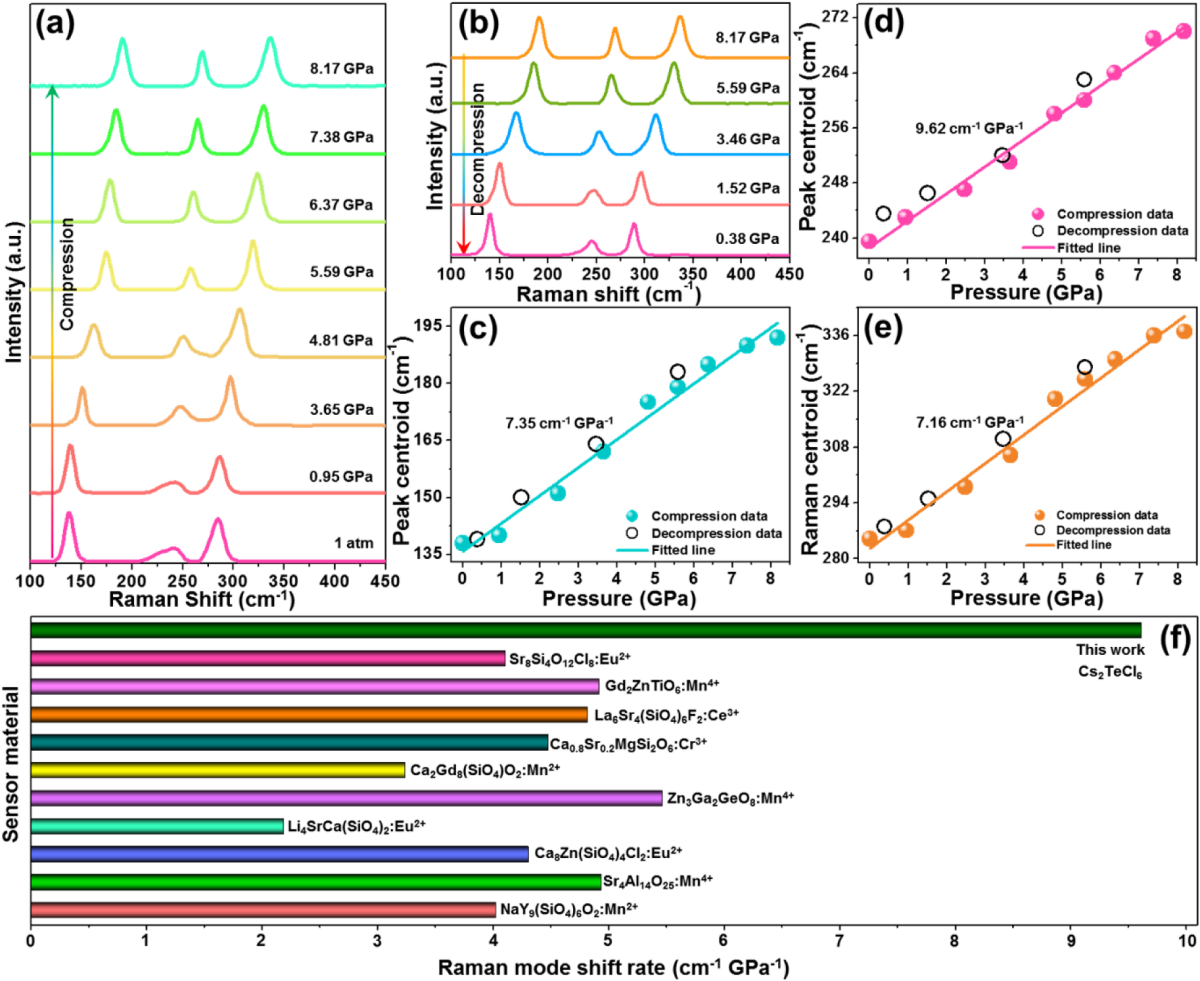
Pressure-related Raman spectra of the Cs_2_TeCl_6_ nanocrystals during (a) compression and (b) decompression processes. (c–e) Calculated Raman peak centroids as a function of pressure. (f) Pressure-triggered Raman mode shift rates (*i.e*., pressure sensitivities) of diversified luminescent pressure sensors reported. These data can be found in refs 4, 7–10, 39, 44–46, and 48 , and the pressure sensitivity of the studied samples in this work belongs to the highest value.

For the sake of exploring the impact of pressure on the luminescence features of the Cs_2_TeCl_6_ material, as well as its feasibility as an optical manometer, its pressure-dependent emission spectra were measured. The experimental setup for measuring the *in* *situ* luminescence properties at high pressure is presented in Figure 4a. Figure 4b shows the normalized pressure-dependent emission spectra of the Cs_2_TeCl_6_ nanocrystals in the range of 0–8.05 GPa, using an excitation wavelength of 375 nm. As disclosed, these recorded emission spectra are all composed of an intense broad band arising from the STE emission. Note that a gradual blue shift (*i.e*., from 596.3 to 581.0 nm) occurs in the emission band as pressure increases Owing to the observed spectral shift, it is found that the resulting nanocrystals exhibit polychromatic luminescence, *i.e*., the emitting color changes from orange to yellow with increasing pressure in the range of 0–8.05 GPa, as displayed in Figure 4c, enabling its application in visual optical pressure sensing. Moreover, the correlated color temperature of the generated emission in Cs_2_TeCl_6_ nanocrystals is also impacted by pressure; namely, it alters from 2183 to 2714 K within the applied pressure range of 0–8.05 GPa, as listed in Table S1. The pressure-related emission band centroid for Cs_2_TeCl_6_ is presented in Figure 4d. Evidently, it can be fitted with a second-order polynomial function, namely, λ = 0.21*p* ^2^ – 3.54*p* + 596.18. To quantitatively describe the manometric properties of the studied sample, the sensitivity (*i.e*., dλ/d*p*) was calculated, and the corresponding results are shown in Figure 4e. It is found that the dλ/d*p* value shows a downward tendency with an increase in pressure, achieving its maximum value of 3.54 nm/GPa at the initial pressure. Here, the determined maximum dλ/d*p* value is not only ≈10 times larger than that for the commonly used optical manometers based on ruby (dλ/d*p* = 0.365 nm GPa^–1^), but also comparable with most of the previously developed optical manometers, which were based on the visible emissions of luminescent materials, as listed in Table 1, demonstrating its application potential in optical pressure monitoring. Aside from the blue shift of the emission band at high pressure, the fwhm is also found to be dependent on pressure; *i.e*., it decreases from 119.1 to 99.9 nm with the pressure increasing from 0 to 8.05 GPa (see Figure 4f). Such a phenomenon is rarely observed, and it can be found in some perovskite materials, which exhibit STE emission. The discussed effect of narrowing of the PL band, associated with exciton emission in the perovskite structure, is triggered by the pressure-induced decrease of the lattice relaxation energy, *i.e*., weakening of the electron–phonon coupling with pressure.^33,46^ Moreover, the relation between fwhm and pressure can also be fitted *via* a polynomial expression, namely, fwhm = 0.28*p* ^2^ – 4.82*p* + 119.53. Accordingly, the pressure-dependent sensitivity based on fwhm, *i.e*., dfwhm/d*p*, was determined and is depicted in Figure 4g. With increasing pressure, a gradual decreasing tendency is seen in the dfwhm/d*p* parameter, with a maximum value of 4.82 nm GPa^–1^. As displayed in Table 1, it is clear that the calculated dfwhm/d*p* value of the Cs_2_TeCl_6_ nanocrystals is superior to that of most fwhm-based optical manometers, implying its feasibility in optical manometry. Furthermore, it is shown in Figure S6 that the emission band centroid and fwhm of the Cs_2_TeCl_6_ nanocrystals can return to their starting states even after two compression–decompression processes, suggesting that the resulting nanocrystals possess good stability and reversibility, which is beneficial for their application in optical pressure sensing.

**Table 1:** Comparison of the Pressure Sensing Capabilities of Previously Reported Optical Manometers with Those of the Designed Nanocrystals Operating in the Visible Range

			Sensitivity		
Compound	Operating range (GPa)	Centroid (nm)	dλ/d*p*	dfwhm/d*p*	Reference
NaY_9_(SiO_4_)_6_O_2_:Mn^2+^	0.75–7.16	617.2	7.00 nm GPa^–1^	10.13 nm GPa^–1^	^4^
Y_6_Ba_4_(SiO_4_)_6_F_2_:Ce^3+^	0–31	466	0.63 nm GPa^–1^	∼1.81 nm GPa^–1^	^6^
Ca_8_Zn(SiO_4_)_4_Cl_2_:Eu^2+^	0.46–20.35	500.2	4.18 nm GPa^–1^	1.63 nm GPa^–1^	^8^
Li_4_SrCa(SiO_4_)_2_:Eu^2+^	0–10	584	5.19 nm GPa^–1^	1.23 nm GPa^–1^	^9^
Zn_3_Ga_2_GeO_8_:Mn^4+^	0–19.9	695	0.844 nm GPa^–1^	-	^10^
Sr_2_[MgAl_5_N_7_]:Eu^2+^	0–10.2	650	5.07 nm GPa^–1^	-	^11^
Ba_3_Lu(BO_3_)_3_:Ce^3+^	0–20.8	485	3.51 nm GPa^–1^	-	^12^
Cs_2_Ag_0.6_Na_0.4_InCl_6_:Bi^3+^	0–4	600	112 nm GPa^–1^	-	^33^
La_3_Mg_2_SbO_9_:Mn^4+^	0–9.48	691	1.2 nm GPa^–1^	0.53 nm GPa^–1^	^47^
Sr_8_Si_4_O_12_Cl_8_:Eu^2+^	0–7.35	497.3	9.69 nm GPa^–1^	-	^48^
Cs_2_TeCl_6_	0–8.05	596.3	3.54 nm GPa^–1^	4.82 nm GPa^–1^	This work

**Figure 4:**
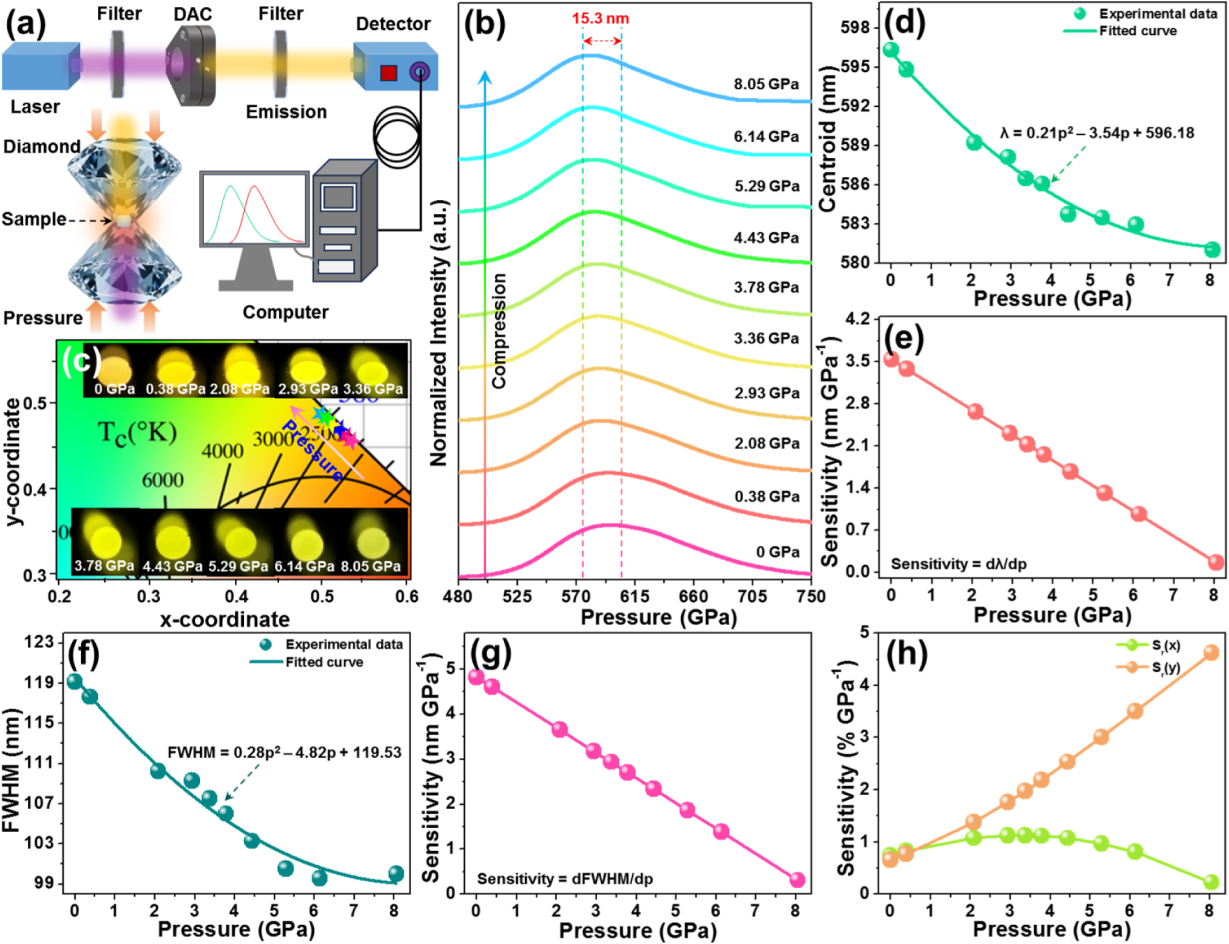
(a) Experimental setup for measuring the pressure-dependent emission spectra. (b) Emission spectra, (c) emitting-color, (d) emission peak centroid, (e) sensitivity (*i.e*., dλ/d*p*), (f) fwhm, (g) sensitivity (*i.e*., dfwhm/d*p*), and (h) *S*_r_(*x*) and *S*_r_(*y*) values of the Cs_2_TeCl_6_ nanocrystals plotted as a function of pressure.

As clarified above, the observed broadband emission of the Cs_2_TeCl_6_ nanocrystals originates from the exciton self-trapping due to the strong coupling between the lattice and exciton, where an obvious spectral blue shift is observed when the sample undergoes the compression process. From previous literature reports,^49,50^ one knows that the blue shift of the STE emission is related to the reduced lattice relaxation energy (*E*_LR_). Particularly, the *E*_LR_ value is proportional to the fwhm of the STE emission, as described below:^49,50^

9 $$\mathrm{fwhm}\propto \sqrt{2E_{\mathrm{LR}}kT}$$

Since the fwhm decreases as the pressure increases (Figure 4f), it can be concluded that the *E*_LR_ value should decrease at high pressure, resulting in the blue shift of the emission band. Furthermore, the electron–phonon coupling strength can be described by using the *S*, where the relation between Stokes shift energy (*i.e*., *E*_stokes_) and *S* can be defined as follows:^36,49^

10 $$E_{\mathrm{stokes}}=2S\hbar \omega_{\mathrm{LO}}$$

where h̅ω_LO_ refers to the energy of longitudinal optical phonons. As for double perovskite, the scattering mechanism through coupling with lattice vibrations is dominated by the longitudinal optical phonon with A_1g_ symmetry. According to the pressure-dependent emission and Raman spectra, it is evident that *E*_stokes_ decreases as pressure rises, while the h̅ω_LO_ value is promoted at high pressure. As a consequence, the *S* value will decrease, which means weakened electron–phonon coupling strength during the compression process, leading to reduced lattice deformation energy and self-trapping energy.^36^ Thereby, the energy of the STE emission improves, resulting in a pressure-triggered spectral blue shift of the Cs_2_TeCl_6_ material. Additionally, the high-pressure-induced octahedral contraction and shortened bond length may also contribute to the spectral blue shift.^36,49,50^ Under the synergistic effects of these factors, the emission band of the Cs_2_TeCl_6_ nanocrystals exhibits a blue shift at elevated pressure.

As displayed in Figure 4c and Table S1, it can be seen that the color coordinates of the Cs_2_TeCl_6_ nanocrystals are sensitive to pressure. On the basis of the previous reports,^8,39^ one knows that the evolution degree of the color coordinate can be characterized *via* the chromaticity shift, *i.e*., Δs, as defined below:

11 $$\Delta s=\sqrt{{\left(u_{m}^{\prime }-u_{0}^{\prime }\right)}^{2}+{\left(v_{m}^{\prime }-v_{0}^{\prime }\right)}^{2}+{\left(w_{m}^{\prime }-w_{0}^{\prime }\right)}^{2}}$$

where *u* ^´^ = 4*x*/(3 – 2*x* + 12*y*), *v* ^*´*^ = 9*y*/(3 – 2*x* + 12*y*), *w* ^*´*^ = 1 – *u*′– *v*′, and the color coordinates at measured pressure and initial pressure are labeled as *m* and 0, respectively. Within the applied pressure range of 0–8.05 GPa, it is found that the Δ*s* value increases monotonously, *i.e*., changes from 0 to 0.043, as presented in Figure S7. Owing to the obvious shift of the color coordinate, optical pressure detection can be achieved through studying the pressure-dependent color coordinates. It is shown in Figure S8a that the *x*-coordinate increases as pressure elevates, while the *y*-coordinate shows an opposite changing tendency (Figure S8b). Moreover, the relation between the color coordinates and pressure can be fitted by a cubic third-order polynomial expression, that is, *x* = 6.452 × 10^–5^ *p* ^3^ – 6.004 × 10^–4^ *p* ^2^ – 0.004*p* + 0.537 and *y* = −6.851 × 10^–5^ *p* ^3^ + 6.139 × 10^–4^ *p* ^2^ + 0.003*p* + 0.456. For the purpose of evaluating the manometric properties of the nanocrystals studied operating in this mode, the relative sensitivity (*i.e*., *S*_r_) is estimated by means of the following equation:^8,39^

12 $$S_{r}=\left|\frac{1}{C}\frac{dC}{dp}\right|\times 100\%$$

where the *x*-coordinate and *y*-coordinate are attributed to *C*. Note that when the *x*-coordinate and *y*-coordinate are used as the manometric parameters, their relative sensitivities are labeled by *S*_r_(*x*) and *S*_r_(*y*), respectively. Through utilizing the above formula and the fitted results, the pressure-dependent *S*_r_(*x*) and *S*_r_(*y*) values are obtained and presented in Figure 4h. As disclosed, the *S*_r_(*y*) value increases gradually as pressure rises, achieving its maximum value of 4.62% GPa^–1^, while the *S*_r_(*x*) value exhibits a diverse changing tendency, and its maximum value is 1.12% GPa^–1^, demonstrating that colorimetric pressure sensing can be realized in the resulting sample. These achievements indicate that the Cs_2_TeCl_6_ nanocrystals with high sensitivities and good stability are promising candidates for multiparameter optical manometers through employing various parameters of emission band centroid, fwhm, and color coordinate.

## 4 Conclusions

In summary, the Cs_2_TeCl_6_ nanocrystals were prepared to develop a bifunctional and multiparameter, highly sensitive optical thermometer and manometer. Excited at 456 nm, the resulting nanocrystals can emit intense broadband emission centered at 596 nm, pertaining to the STE emission. To explore the temperature-sensing capabilities of the designed compound, temperature-dependent decay curves were measured. Within the applied temperature range (*i.e*., 183–303 K), the decay time shortens gradually and the maximum *S*_r_ value is determined to be 3.76% K^–1^ at 263 K. Moreover, for the aim of investigating the potential application of the Cs_2_TeCl_6_ nanocrystals in optical pressure detection, *in situ* high-pressure Raman and emission spectra were examined. The pressure-dependent Raman spectra indicate that the studied sample possesses splendid structural stability and reversibility within the pressure range of 1 atm −8.17 GPa. When the Raman mode is adopted as the manometric parameter, the pressure sensitivity of the Cs_2_TeCl_6_ nanocrystals is 9.62 cm^–1^ GPa^–1^. Furthermore, as pressure increases, a significant spectral blue shift is seen in the Cs_2_TeCl_6_ spectra, resulting in pressure-induced color-tunable emissions, *i.e*., from orange to yellow. By using the emission band centroid and fwhm as manometric parameters, the maximum pressure sensitivities for the Cs_2_TeCl_6_ material are determined to be 3.54 and 4.82 nm GPa^–1^, respectively. Additionally, colorimetric pressure sensing is also implemented for the synthesized nanocrystals, of which the maximum sensitivity is 4.62% GPa^–1^ when the *y*-coordinate is adopted as the manometric parameter. These results manifest that the luminescence characteristics of the Cs_2_TeCl_6_ nanocrystals can be regulated *via* high-pressure and low-temperature stimuli, enabling their promising application in highly sensitive optical thermometry and manometry.

## Supplementary Material



## Notes

First-principles density function calculation details, color coordinates, and correlated color temperature of the Cs_2_TeCl_6_ nanocrystals as a function of pressure; XRD profile, XPS spectra, diffuse reflectance spectrum, and decay curve of the Cs_2_TeCl_6_ nanocrystals; XRD patterns of the Cs_2_TeCl_6_ nanocrystals at different temperatures; pressure-dependent emission spectra; emission band centroid, fwhm, and Δ*s* values of the Cs_2_TeCl_6_ nanocrystals; *x*-coordinate and *y*-coordinate of the Cs_2_TeCl_6_ nanocrystals as a function of pressure (PDF)
