# Supplementary material for: Tailoring the Luminescence Properties of Cs 2 TeCl 6 Nanocrystals via High-Pressure and Low-Temperature Stimuli for Multiparameter Optical Manometry and Thermometry
Source: Inorg Chem. 2025 Sep 26;64(40):20439–48. doi: 10.1021/acs.inorgchem.5c03775 (PMC12522140; doi:10.1021/acs.inorgchem.5c03775)
Supplement: Supplementary file 1 [file ic5c03775_si_001.pdf]

Tailoring the luminescence properties of Cs_2TeCl_6 nanocrystals via high-pressure and low-temperature stimuli for multi-parameter optical manometry and thermometry

Zhiyu Pei[a], Marcin Runowski[b*], Przemysław Woźny[b], Peng Du[a*], Ning Chen[a*]

[a]*School of Physical Science and Technology, Ningbo University, 315211 Ningbo, Zhejiang, China*

[b]*Adam Mickiewicz University, Faculty of Chemistry, Uniwersytetu Poznańskiego 8, 61-614 Poznań, Poland*

Corresponding authors:

E-mail: runowski@amu.edu.pl (M. Runowski); dupeng@nbu.edu.cn or dp2007good@sina.com (P. Du); chenning@nbu.edu.cn (N. Chen)

First-principles density function calculation

The first-principles density function (DFT) calculation using the Cambridge Sequential Total Energy Package code (CASTEP) was performed so as to understand the electronic structure of the prepared product,[1-3] of which the crystal was modelled via a periodic 2 × 2 × 2 supercell. For the sake of ensuring the accuracy of the calculation results, we used the Vanderbilt ultrasoft pseudopotential, in which the cutoff energy was set as 450 eV. To optimize the geometry and lattice size, the Brillouin zone integration was performed with 3 × 3 × 3 Γ-centered k-point sampling.[4] Furthermore, to optimize the geometry, the Broyden, Fletcher, Goldfarb, Shannon (BFGS) method was employed. During the whole geometry optimization process, the positions of the atoms were simultaneously optimized. The convergence criteria for geometry

optimization including the following parts: total energy change was 10^{-6} eV per atom, the maximum ionic Hellmann-Feynman force difference was 0.02 eV/Å, stress tensor variation was 2×10^{-2} GPa and maximum atomic displacement was 5×10^{-4} Å. Herein, the calculation was carried out within the framework of generalized gradient approximation, incorporating exchange and correlation functions. The self-consistent field convergence criterion was consistently maintained at 1.0×10^{-5} eV per atom during the entire computational process.

Table S1 Color coordinate and CCT values of the Cs_2TeCl_6 nanocrystals as a function of pressure.

Pressure	Color coordinate		CCT (K)
	x	*y*	
0 GPa	0.53778	0.45576	2183
0.38 GPa	0.53624	0.45773	2207
2.08 GPa	0.52633	0.46705	2348
2.93 GPa	0.5253	0.46803	2363
3.36 GPa	0.5218	0.47123	2415
3.78 GPa	0.51902	0.47382	2458
4.43 GPa	0.51216	0.48006	2563
5.29 GPa	0.51088	0.4817	2586
6.14 GPa	0.50817	0.4841	2628
8.05 GPa	0.50237	0.48835	2714

Calculation of CCT value

The correlated color temperature (CCT) values of the generated emissions in the Cs_2TeCl_6 nanocrystals were calculated through the following expression:[5]

$$\mathrm{CCT} = -437n^3 + 3601n^2 - 6846n + 5514.31 \quad \text{(S1)}$$

$$n = (x - x_e)/(y - y_e) \quad \text{(S2)}$$

here (x_e,y_e) = (0.3320,0.1858) and (x,y) is assigned to the color coordinates of the generated emissions. By means of the aforementioned formula and the calculated color coordinates, the CCT values of the Cs_2TeCl_6 nanocrystals as a function of pressure were estimated and displayed in Table S1. Significantly, the CCT values of the resulting samples are increased from 2183 to 2714 K with the increment of pressure.

References

[1] Hohenberg, P.; Kohn, W. Inhomogeneous electron gas, *Phys. Rev*. **1964**, *136*, B864-B871.

[2] Kohn, W.; Sham, L. J. Self-consistent equations including exchange and correlation effects, *Phys. Rev*. **1965**, *140*, A1133-A1138.

[3] Payne, M. C.; Teter, M. P.; Allan, D. C.; Arias, T.; Joannopoulos, J. Iterative minimization techniques for ab initio total-energy calculations: molecular dynamics and conjugate gradients, *Rev. Mod. Phys*. **1992**, *64*, 1045-1097.

[4] Monkhorst, H. J.; Pack, J. D. Special points for Brillouin-zone integrations, *Phys Rev B* **1976**, *13*, 5188-5192.

[5] Zhang, M.; Guo, X.; Cui, R.; Zhang, J.; Deng, C. A thermally stable color-tunable

white phosphor $CaGa_{0.5}Nb_{0.5}O_3:Dy^{3+}/Sm^{3+}$ for warm WLEDs, *Ceram. Int.* **2025**, *51*, 9740-9752.

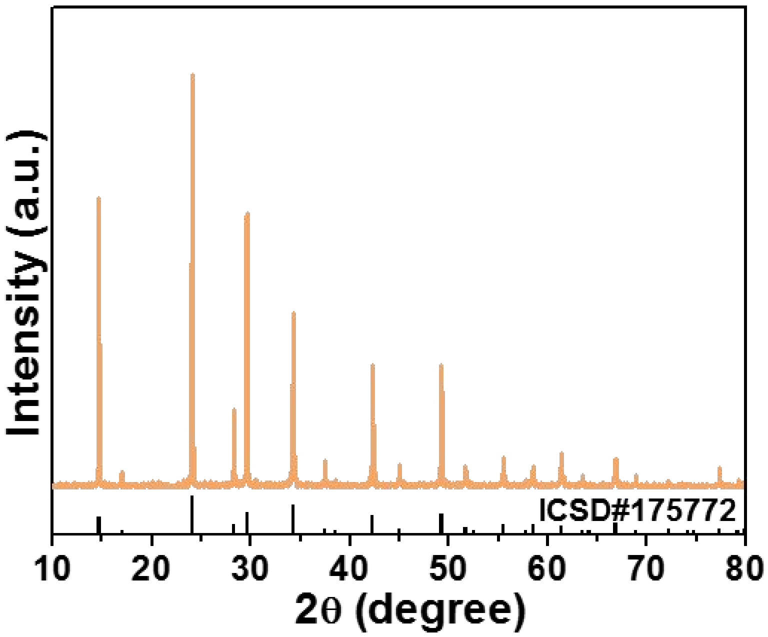

Figure S1 XRD profile of the Cs_2TeCl_6 nanocrystals.

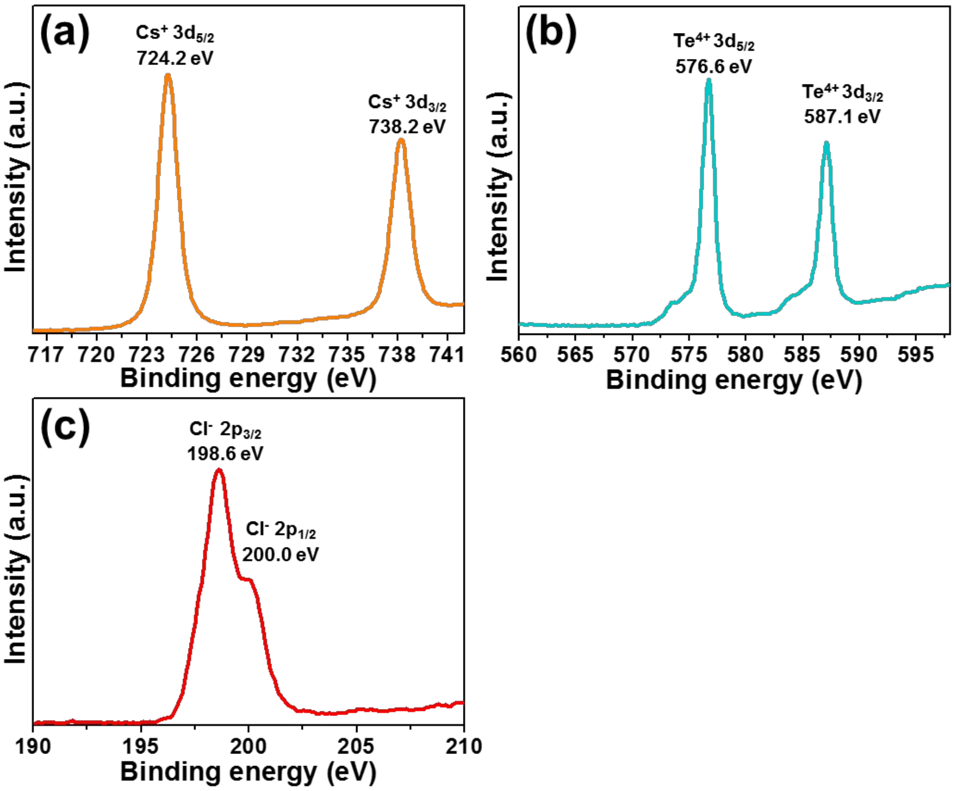

Figure S2 High-resolution XPS spectra of (a) Cs^+ 3d, (b) Te^{4+} 3d and (c) Cl^- 2p in the Cs_2TeCl_6 nanocrystals.

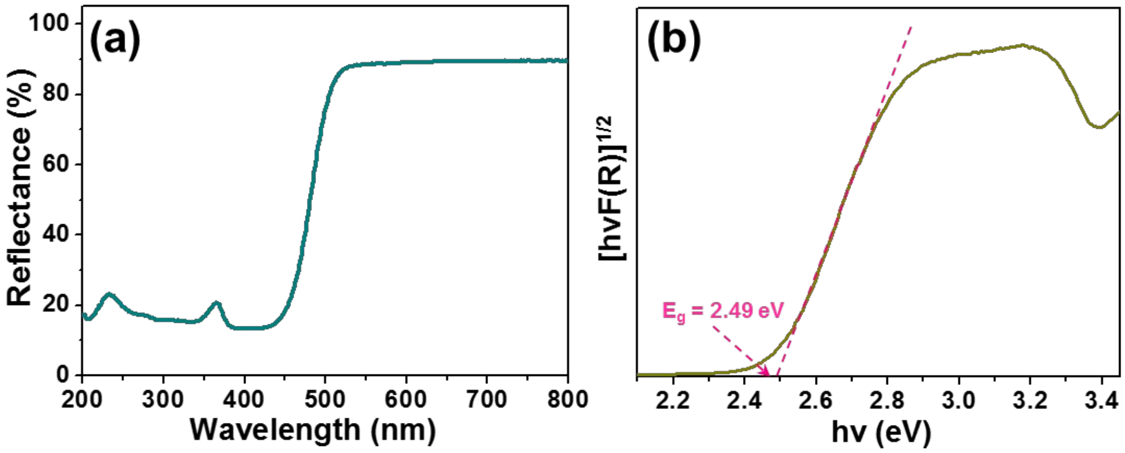

Figure S3 (a) Diffuse reflectance spectrum and (b) calculated E_g value of the Cs_2TeCl_6 nanocrystals.

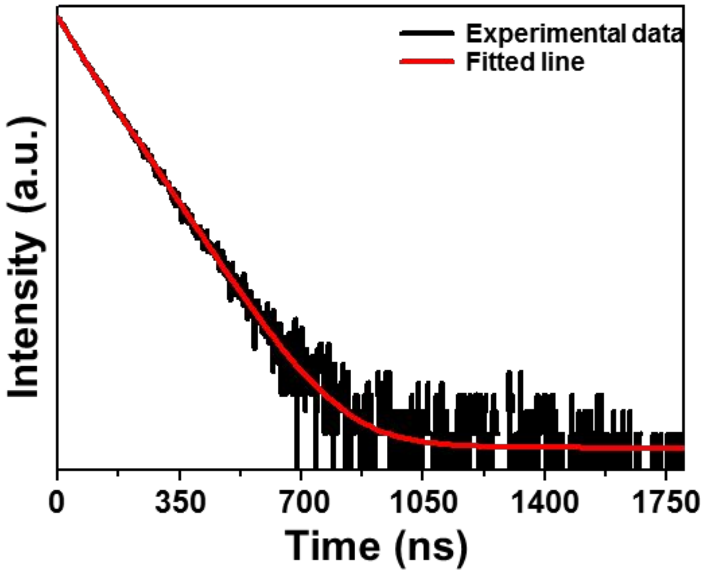

Figure S4 Decay curve of the Cs_2TeCl_6 nanocrystals at room temperature.

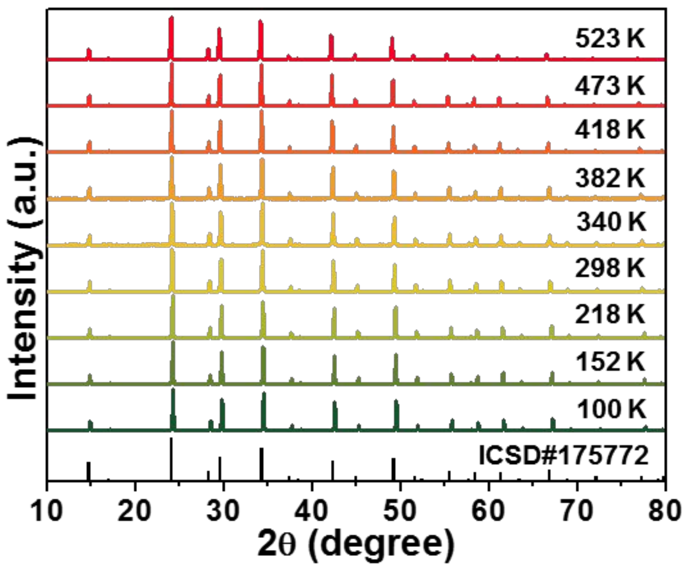

Figure S5 XRD patterns of the Cs_2TeCl_6 nanocrystals as a function of temperature.

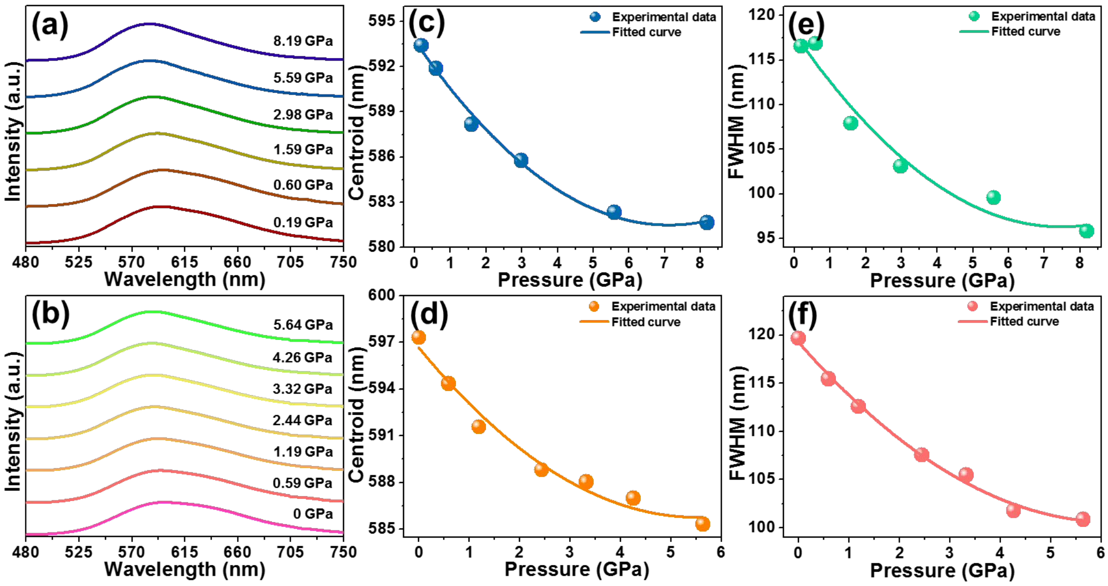

Figure S6 Pressure-dependent (a) and (b) emission specta, (c) and (d) emission band centroid as well as (e) and (f) FWHM values during the compression processes.

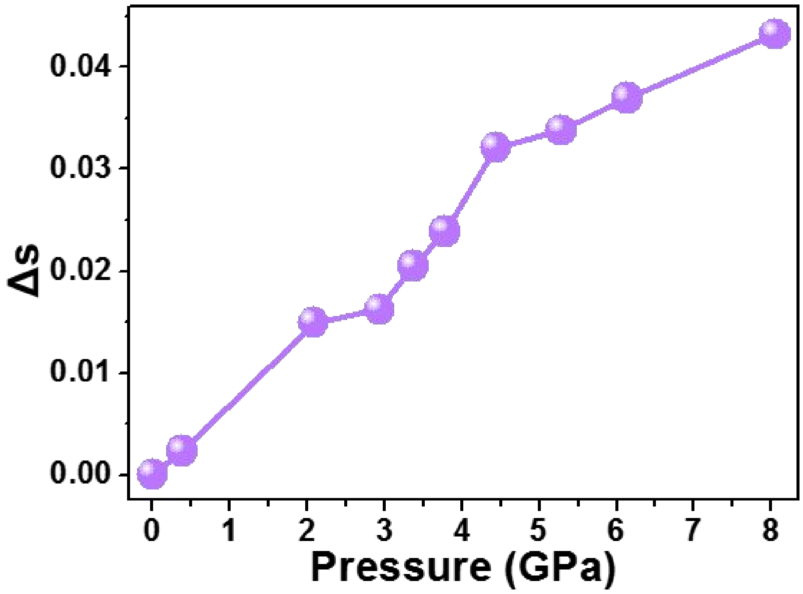

Figure S7 Δs value of the Cs_2TeCl_6 nanocrystals as a function of pressure.

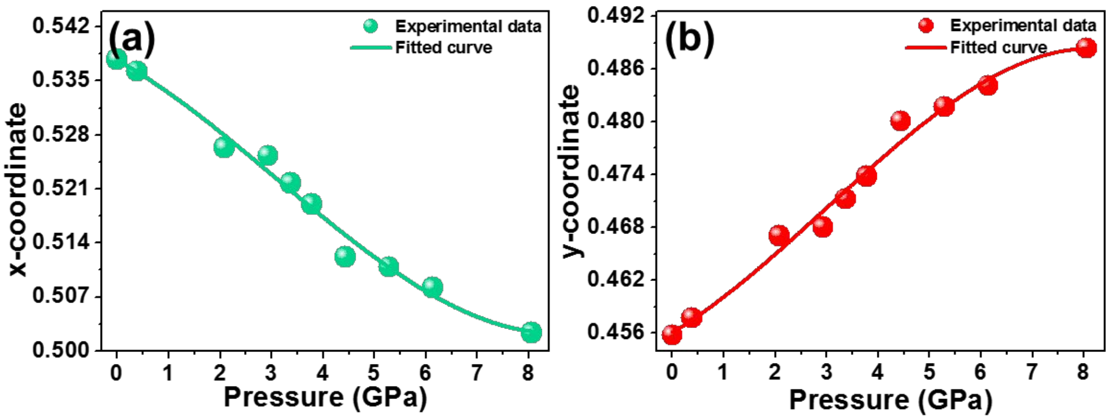

Figure S8 (a) *x*-coordinate and (b) *y*-coordinate of the Cs_2TeCl_6 nanocrystals as a function of pressure.
